# The recently identified flavivirus Bamaga virus is transmitted horizontally by *Culex* mosquitoes and interferes with West Nile virus replication *in vitro* and transmission *in vivo*

**DOI:** 10.1371/journal.pntd.0006886

**Published:** 2018-10-24

**Authors:** Agathe M. G. Colmant, Sonja Hall-Mendelin, Scott A. Ritchie, Helle Bielefeldt-Ohmann, Jessica J. Harrison, Natalee D. Newton, Caitlin A. O’Brien, Chris Cazier, Cheryl A. Johansen, Jody Hobson-Peters, Roy A. Hall, Andrew F. van den Hurk

**Affiliations:** 1 Australian Infectious Diseases Research Centre, School of Chemistry and Molecular Biosciences, The University of Queensland, St Lucia, QLD, Australia; 2 Public Health Virology, Forensic and Scientific Services, Department of Health, Queensland Government, Coopers Plains, QLD, Australia; 3 College of Public Health, Medical and Veterinary Sciences, James Cook University, Cairns, QLD, Australia; 4 Australian Institute of Tropical Health and Medicine, James Cook University, Cairns, QLD, Australia; 5 School of Veterinary Science, The University of Queensland, Gatton Campus, QLD, Gatton Australia; 6 Technical Services, Biosciences Division, Faculty of Health, Queensland University of Technology, Gardens Point Campus, Brisbane, Qld, Australia; 7 PathWest Laboratory Medicine WA, Nedlands, Western Australia, Australia; 8 School of Biomedical Sciences, The University of Western Australia, Nedlands, Western Australia, Australia; University of Texas Medical Branch, UNITED STATES

## Abstract

Arthropod-borne flaviviruses such as yellow fever (YFV), Zika and dengue viruses continue to cause significant human disease globally. These viruses are transmitted by mosquitoes when a female imbibes an infected blood-meal from a viremic vertebrate host and expectorates the virus into a subsequent host. Bamaga virus (BgV) is a flavivirus recently discovered in *Culex sitiens* subgroup mosquitoes collected from Cape York Peninsula, Australia. This virus phylogenetically clusters with the YFV group, but is potentially restricted in most vertebrates. However, high levels of replication in an opossum cell line (OK) indicate a potential association with marsupials. To ascertain whether BgV could be horizontally transmitted by mosquitoes, the vector competence of two members of the *Cx*. *sitiens* subgroup, *Cx*. *annulirostris* and *Cx*. *sitiens*, for BgV was investigated. Eleven to thirteen days after imbibing an infectious blood-meal, infection rates were 11.3% and 18.8% for *Cx*. *annulirostris* and *Cx*. *sitiens*, respectively. *Cx*. *annulirostris* transmitted the virus at low levels (5.6% had BgV-positive saliva overall); *Cx*. *sitiens* did not transmit the virus. When mosquitoes were injected intrathoracially with BgV, the infection and transmission rates were 100% and 82%, respectively, for both species. These results provided evidence for the first time that BgV can be transmitted horizontally by *Cx*. *annulirostris*, the primary vector of pathogenic zoonotic flaviviruses in Australia. We also assessed whether BgV could interfere with replication *in vitro*, and infection and transmission *in vivo* of super-infecting pathogenic *Culex-*associated flaviviruses. BgV significantly reduced growth of Murray Valley encephalitis and West Nile (WNV) viruses *in vitro*. While prior infection with BgV by injection did not inhibit WNV super-infection of *Cx*. *annulirostris*, significantly fewer BgV-infected mosquitoes could transmit WNV than mock-injected mosquitoes. Overall, these data contribute to our understanding of flavivirus ecology, modes of transmission by Australian mosquitoes and mechanisms for super-infection interference.

## Author summary

Mosquito-borne flaviviruses include medically significant members such as the dengue viruses, yellow fever virus and Zika virus. These viruses regularly cause outbreaks globally, notably in tropical regions. The ability of mosquitoes to transmit these viruses to vertebrate hosts plays a major role in determining the scale of these outbreaks. It is essential to assess the risk of emergence of flaviviruses in a given region by investigating the vector competence of local mosquitoes for these viruses. Bamaga virus was recently discovered in Australia in *Culex* mosquitoes and shown to be related to yellow fever virus. In this article, we investigated the potential for Bamaga virus to emerge as an arthropod-borne viral pathogen by assessing the vector competence of *Cx*. *annulirostris* and *Cx*. *sitiens* mosquitoes for this virus. We showed that Bamaga virus could be detected in the saliva of *Cx*. *annulirostris* after an infectious blood-meal, demonstrating that the virus could be horizontally transmitted. In addition, we showed that Bamaga virus could interfere with the replication *in vitro* and transmission *in vivo* of the pathogenic flavivirus West Nile virus. These data provide further insight on how interactions between viruses in their vector can influence the efficiency of pathogen transmission.

## Introduction

The genus *Flavivirus* encompasses over 70 viral species including several human and animal pathogens, such as yellow fever virus (YFV), dengue viruses (DENV), Zika virus (ZIKV), West Nile virus (WNV) and Murray Valley encephalitis virus (MVEV) which are transmitted by mosquitoes [1, 2]. Even though most flaviviruses can replicate in *Aedes*, *Culex*, *or Anopheles* cells *in vitro* and sometimes also *in vivo*, flaviviruses are thought to be either *Culex-* (WNV, MVEV) or *Aedes-*associated (DENV, ZIKV, YFV) in relation to their main vector for transmission [3–5]. Horizontal transmission of these arthropod-borne viruses (arboviruses) occurs when the virus is ingested by a mosquito whilst it feeds on infected blood from a vertebrate host. Post ingestion by the mosquito, the virus infects and replicates in the midgut epithelial cells [6, 7]. The virus then disseminates from the midgut cells and typically undergoes secondary replication in other tissues, such as fat bodies or neural tissues. Finally, the virus infects the cells of the salivary glands before being released into the salivary secretion when the mosquito probes a vertebrate host during feeding [6, 7]. Several barriers to infection within the mosquito must be overcome before transmission of an arbovirus including the midgut infection and escape barriers, and the salivary infection and escape barriers [8, 9]. Vector-competence studies aim to determine if a mosquito species can transmit an arbovirus, by evaluating if and how well the virus can overcome the infection, dissemination and transmission barriers in those mosquitoes [10–17]. These laboratory-based studies producing vectorial capacity data are crucial to determine whether these viruses pose a threat of an epidemic transmission by local mosquito species, or simply to better understand the ecological niches in which these viruses belong.

Bamaga virus (BgV) is a flavivirus which was recently isolated from archival samples of *Cx*. *annulirostris* mosquitoes collected in 2001 and 2004 in Cape York, Far North Queensland, Australia [18]. While BgV is phylogenetically most closely related to the Australian flavivirus Edge Hill virus and other vertebrate-infecting members of the YFV group, initial *in vitro* characterisation experiments indicated that BgV was not able to replicate in a range of vertebrate cell lines (monkey, chicken, rabbit) suggesting it may have a restricted or narrow vertebrate host range [18]. In addition, injecting the virus in mice produced no disease and only caused signs of replication-associated pathology when the highest dose of virus was injected directly into the brain of the animals [18]. Despite this attenuation, BgV is classified as a vertebrate-infecting flavivirus based on its phylogenetic position, its ability to replicate to low levels in selected vertebrate cell lines (hamster, opossum, human), and its dinucleotide usage bias [18, 19]. To determine whether the virus could be horizontally transmitted by mosquitoes, laboratory-based experiments were conducted to assess BgV infection, dissemination and transmission rates in *Cx*. *annulirostris* and the closely related *Cx*. *sitiens*.

Virus co- and super-infection are defined by the simultaneous or sequential infection of cells, animals or mosquitoes by two different viruses. It has been shown for a number of vertebrate-infecting flaviviruses that the level of replication or transmission of a co- or super-infecting flavivirus could be regulated by the presence of the first, both *in vitro* and *in vivo* [20]. Examples of this phenomenon include Bagaza virus which suppressed replication of Japanese encephalitis virus (JEV) and WNV in *Culex* mosquitoes upon co- and super-infection [21]; WNV and SLEV which could inhibit replication and dissemination of one another *in vivo* [22]; and DENV and YFV which could suppress replication of the other *in vitro* [23]. Furthermore, there is a subset of flaviviruses that only infect insects, and therefore, have no vertebrate hosts, but have been thoroughly studied in recent years because of their potential for co- or super-infection interference with pathogenic vertebrate-infecting flaviviruses and their high prevalence in certain mosquito populations [24, 25]. For instance, it has been shown that WNV replication (*in vitro* and *in vivo)* and transmission by *Culex* mosquitoes could be regulated by the presence of the insect-specific flavivirus, Palm Creek virus [26, 27]. Such interactions are important to understand in the context of risk assessment of the likelihood of an arbovirus being transmitted by local populations of mosquitoes. To further explore the phenomenon of competitive interference, we also assessed whether the presence of BgV in mosquito cells and in live mosquitoes could interfere with the replication or transmission of *Culex-*associated medically significant flaviviruses.

## Material and methods

### Cell culture

C6/36 cells (*Ae*. *albopictus*) were cultured in Roswell Park Memorial Institute 1640 while Vero cells (*Cercopithecus aethiops*, African green monkey, kidney epithelial cells) were cultured in Dulbecco’s Modified Eagle’s Medium. Both cell culture media were supplemented with 2–10% fetal bovine serum (FBS), 50U penicillin/mL, 50μg streptomycin/mL and 2mM L-glutamine.

### Screening mosquito homogenates

Archival and recent mosquito homogenates were screened for the presence of BgV using the broad-spectrum Monoclonal Antibodies to Viral RNA Intermediates in Cells (MAVRIC) detection system [28]. Briefly, mosquitoes were collected in the wild using CO_2_ baited light traps as described previously [29]. Collections were sorted and female mosquitoes identified to species or genus level, pooled and homogenised in cell culture medium using glass beads and a Tissue Lyser III (Qiagen) for three minutes at 30Hz or following previously published methods [30]. The homogenates were clarified by centrifugation at 12,000 g for five minutes and filtered through a 0.2/0.8 μm sterile filter. The filtered homogenates were then inoculated on four wells of a 96 well plate pre-seeded with C6/36 mosquito cells and incubated at 28°C for 5-7days. After incubation, the cell supernatant was harvested and stored at -80°C, the cells were fixed and tested in fixed-cell enzyme-linked immunosorbent assay (ELISA) as described below using anti-dsRNA monoclonal antibodies MAVRIC, or pan-flavivirus monoclonal antibody (mAb) 4G2. RNA was extracted from the harvested supernatant of positive samples using the Nucleospin Viral RNA extraction kit (Macherey Nagel) following the manufacturer’s instruction and tested by reverse-transcription PCR (RT-PCR) using pan-flavivirus primers and the Superscript III and Platinum Taq One-step RT-PCR kit (Invitrogen) following the manufacturer’s instructions [31].

### Fixed-cell ELISA

Fixed cells were blocked for 30 minutes at room temperature (RT) in blocking buffer (0.05 M Tris/HCl (pH 8.0), 1 mM EDTA, 0.15 M NaCl, 0.05% (v/v) Tween-20, 0.2% w/v casein). Primary mAb, at the optimal dilution in blocking buffer, was added to each well after removing the blocking buffer and incubated at 37°C for one hour. Plates were washed with PBS containing 0.05% Tween-20 (PBS-T) four times and secondary horse radish peroxidase-conjugated antibody (goat anti-mouse, Dako) was added diluted 1/3000 in blocking buffer and incubated at 37°C for one hour. Plates were washed six times with PBS-T and ABTS based substrate (1mM 2,2'-azino-bis(3-ethylbenzothiazoline-6-sulphonic acid) with 3mM hydrogen peroxide in a 0.1M citrate / 0.2M Na_2_PO_4_ buffer pH 4.2) was added and left to develop in the dark at RT for one hour. Finally, the absorbance of each well was measured by an automated 96-well spectrophotometer at 405 nm. Positive wells were identified with a threshold of optical density higher than twice the average of mock infected wells.

### Virus culture for *in vitro* and *in vivo* experiments

The virus strains used were BgV prototype CY4270 (stock with passage number 6, passaged only in C6/36 cells) [18], WNV New South Wales 2011 strain (passaged on C6/36, Vero and C6/36 cells successively) [32], MVEV strain 1–51 [33], and Ross River virus (RRV) strain T-48 [34].

### Virus titration by 50% tissue culture infective dose (TCID_50_) assay

Titrated samples were serially diluted eight times 10-fold on C6/36 or Vero cells in 96 well plates, with four to ten replicate wells per dilution. The plates were incubated for five days at 28°C or 37°C respectively, fixed in 20% acetone, 0.02% bovine serum albumin in PBS, and assessed by fixed-cell ELISA as described above.

### Super-infection interference *in vitro*

C6/36 cells were incubated in suspension, rocking at RT for two hours either in mock cell culture medium or medium containing BgV to obtain a multiplicity of infection of 10 and seeded in a T175 flask to grow at 28°C for five days. After this incubation, cells were reseeded at 5x10^4^ cells per well in 24 well plates in triplicates for each time point and virus, with one extra well seeded onto a glass coverslip, and incubated for two days at 28°C. The mock and BgV coverslips were fixed in ice cold acetone and immunolabeled by immunofluorescence assay using vertebrate-infecting flavivirus E cross-reactive mAb 4G2, as described previously, to confirm BgV infected all the cells [18]. Cells were washed with sterile PBS once and mock- and BgV-infected cells were inoculated with either MVEV, WNV or RRV at a multiplicity of infection of 0.01. After 20 minutes rocking at RT and one hour incubation at 28°C, inoculum was removed, cells washed with sterile PBS thrice and topped up with 750μL of growth medium. Culture supernatants were harvested at 1h, 8h, 24h, 48h and 72h post-infection and titrated by TCID_50_ on Vero cells as described above. After incubation for five days at 37°C, the cells were fixed and analysed by fixed-cell ELISA as described above, using anti-flavivirus non-structural protein 1 mAb 4G4 for MVEV and WNV (non-reactive to BgV), and anti-RRV mAb G8.

### Mosquito maintenance

Mosquitoes were collected from near Cairns (16^o^49’S, 145^o^42’E), north Queensland in April 2016 using CO_2_-baited passive traps and shipped to the insectary at Forensic and Scientific Services, Department of Health, Queensland Government, Brisbane, Australia [35]. Insectary conditions were 26°C and 12:12 light:dark whilst all mosquito adults were provided 15% honey water as a nutrient source. To stimulate egg production, mosquitoes were offered defibrinated sheep’s blood as a blood-meal for two hours with a Hemotek feeding apparatus (Discovery Workshops, Accrington, Lancashire, United Kingdom) and pig’s intestine as membrane. The blood engorged females were sorted by species and placed in 30x30x30 cm cages (BugDorm, MegaView Science Co., Ltd, Taiwan). Mosquitoes were offered blood-meals an additional eight times over 14 days. A polyethylene container containing double distilled water was added to each cage for oviposition. Egg rafts were removed daily, and first and second instar larvae fed a slurry of Tropical Fish flakes (Wardley’s Tropical Fish Food Flakes, The Hartz Mountain Corporation, New Jersey), whilst third and fourth instar larvae were fed on cichlid pellets (Kyorin Co. Ltd, Himeji, Japan). Pupae were removed daily and placed in cages for emergence. Ten to fifteen day old female mosquitoes were used for the BgV vector competence assessment.

Mosquitoes for the virus interference experiments were collected from the suburbs of Oxley (27^o^33’S, 152^o^58’E) and Banyo (27^o^22’S, 153^o^04’E) in Brisbane, using CO_2_-baited light traps. Mosquitoes were transported to the Forensic and Scientific Services insectary and *Cx*. *annulirostris* removed, placed in a cage and used in the experiments within 24 hours of collection.

### Mosquito exposure to BgV for vector competence

Mosquitoes were exposed to BgV *via* feeding on a blood/virus mixture with 10^7^ TCID_50_ IU/mL of virus using a Hemotek feeding apparatus. Mosquitoes were also exposed to virus by intrathoracic inoculation of approximately 220nL of BgV at a titre of 10^5^ TCID_50_ IU/mL *i*.*e*. approximately 22 TCID_50_ IU/mosquito or 3% FBS cell culture media as mock inoculum using a Nanoject II (Drummond Scientific, Broomall, PA) micro injector. Mosquitoes were maintained at 28°C, high humidity and 12:12 dark:light cycle in an environmental growth cabinet (Sanyo Electric, Gunma, Japan), and provided 15% honey water as a nutrient source.

### Harvesting and processing of mosquitoes for vector competence

To assess infection, dissemination and transmission rates, mosquitoes were harvested after incubation for 8–13 days post exposure. Unfortunately, *Cx*. *sitiens* mosquitoes displayed a high mortality rate post-emergence and post-exposure, limiting the numbers available for assessment. A forced salivation method was used to assess transmission potential [36]. Briefly, legs+wings were removed from each mosquito, whose proboscis was then placed in a capillary tube with growth media with 20% FBS for two hours. The saliva samples were dispensed into 600μL of 3% FBS growth media. Bodies and legs+wings were placed separately in 2mL U-bottom tubes containing 1mL of 3% FBS growth media, in order to assess separately for virus infection and dissemination. All samples were stored at -80°C. In order to examine potential tissue tropism of BgV in mosquitoes, *Cx*. *annulirostris* exposed to virus by infectious blood-meal (n = 25) or *via* intrathoracic inoculation (n = 25) were fixed in 4% formaldehyde, 0.05% Triton X-100 (BioRad) in PBS for 24h before legs+ wings were removed and bodies transferred to 70% ethanol for storage.

### Super-infection interference *in vivo*

Mosquitoes were CO_2_ anaesthetized, immobilised on a refrigerated table, and injected with approximately 220nL of BgV stock virus diluted in growth medium with 3% FBS to provide a final titre of approximately 10^5^ TCID_50_ IU/mL *i*.*e*. approximately 22 TCID_50_ IU/mosquito. Control mosquitoes were injected with growth medium only. After 7–8 days incubation at 28°C, 12:12 light:dark cycle and high relative humidity, mosquitoes were offered a blood-meal containing 10^6^ TCID_50_ IU/mL of WNV. Mosquitoes were again incubated at 28°C, 12:12 dark:light cycle and high relative humidity, before bodies, legs+wings and saliva expectorates were collected seven or ten days post-blood-meal as described above, and stored at -80°C.

### Virus detection and quantification

Bodies and legs+wings samples from the vector competence study were homogenised with a metal bead in a Tissue Lyser III (Qiagen) for three minutes at 30Hz, clarified by centrifugation at 12,000 g for five minutes and filtered through a 0.22μm sterile filter. Mosquito body homogenates were titrated by TCID_50_ on C6/36 cells as described above. Undiluted supernatant from homogenised legs+wings of positive mosquito bodies were directly inoculated on C6/36 cells, in four wells of a 96 well plate. Finally, saliva expectorates from mosquitoes positive for dissemination (virus detected in legs+wings) were titrated to determine a virus titre in the saliva. Replication was assessed by fixed-cell ELISA (see above) with pan-flavivirus mAb 4G2 [37].

Additionally, 31 BgV injected *Cx*. *annulirostris* mosquitoes were harvested as whole mosquitoes, to be included in the overall infection rate, and homogenised as described above. These were tested for presence of BgV by directly inoculating homogenate on C6/36 cells as described above for the legs+wings and performing a fixed-cell ELISA with mAb 4G2.

Bodies of BgV-injected and mock-injected mosquitoes from the virus interference experiments were processed similarly as above, titrated on Vero cells and analysed by fixed-cell ELISA with mAb 4G4 (WNV reactive and BgV non-reactive). Vero cells were used here to prevent BgV from interfering with WNV replication further, since these vertebrate cells do not support BgV replication. To assess the infection status of the BgV-injected mosquitoes, these homogenates were also inoculated in four wells of a 96 well plate of C6/36 cells and analysed by fixed-cell ELISA with mAb 1B7, which is BgV reactive and WNV non-reactive. The samples were not titrated on C6/36 cells as the potential presence of WNV in the homogenates could have interfered with BgV and altered the titres obtained. Legs+wings from WNV positive bodies were inoculated in four wells of 96 well plates pre-seeded with C6/36 cells, and Vero cells incubated at 28 or 37°C, respectively, for six days before being analysed by fixed-cell ELISA with either mAb 4G4 (Vero) or 1B7 (C6/36) to test for presence of either WNV or BgV, respectively. Similar to body homogenates, saliva from mosquitoes with positive bodies were titrated on Vero cells and inoculated on C6/36 cells as described above and analysed by fixed-cell ELISA with either mAb 4G4 or 1B7.

### BgV localisation in mosquitoes by immunohistochemistry

Fixed mosquitoes were paraffin-embedded prior to immunohistochemistry (IHC) as per routine processing described previously [19]. Five μm sections, collected on charged slides, were immuno-labelled for BgV using a cocktail of BgV reactive mAbs (e. g. 4G2 [38], 6B6C [39], 1D1 [40] and 1B7 [41]) or BgV-specific mouse serum [18] following a previously described protocol [26]. The mAb hybridoma supernatants used in this protocol were tested in fixed-cell ELISA with cells fixed with 4% formaldehyde in PBS with 0.05% Triton X-100 to empirically determine the optimal dilutions to use on the formaldehyde fixed mosquitoes in IHC.

### Analyses

The titres obtained by TCID_50_ were determined using Reed and Muench’s guidelines [42]. For the BgV vector competence experiments, the titre of virus in bodies and saliva expectorates of injected and bloodfed *Cx*. *annulirostris* and *Cx*. *sitiens* were compared using an unpaired parametric t-test. The MVEV, WNV and RRV titres in the *in vitro* super-infection experiment were analysed using an unpaired parametric t-test. For the *in vivo* virus interference experiments, Fisher’s exact tests were used to compare WNV infection, dissemination and transmission rates between BgV-infected and mock-infected *Cx*. *annulirostris*. The titres of WNV positive body samples were statistically analysed using an unpaired parametric t-test. The titres of WNV positive saliva could not be statistically analysed considering that one of the groups only had one positive sample. All analyses were conducted using Graphpad Prism Version 7 (GraphPad Software, Inc, San Diego, USA).

## Results

### BgV ecological niche

There are only three known isolates of BgV, all detected in *Cx*. *sitiens* subgroup mosquitoes collected on Cape York Peninsula, Far North Queensland, Australia between 2001 and 2004 [18]. To further determine the prevalence of BgV in *Culex* and other species in other genera, 811 additional mosquito pools (pool size ranging from 1 to 107) from the *Aedeomyia*, *Aedes*, *Anopheles*, *Coquillettidia*, *Culex*, *Culiseta*, *Mansonia*, *Uranotaenia* and *Verrallina* genera, encompassing at least 31 species, were screened for BgV (Table 1). The mosquito homogenates were inoculated onto C6/36 mosquito cells for isolation and screened using anti-dsRNA mAbs MAVRIC and/or using pan-flavivirus mAb 4G2 in ELISA and/or RT-PCR using pan-flavivirus primers [31]. No BgV isolates were recovered from this range of mosquito homogenates (Table 1).

**Table 1:** Summary of mosquito pools screened for BgV in Australasia. (NT = Northern Territory, Australia; PNG = Papua New Guinea; QLD: Queensland, Australia; WA = Western Australia).

Species	Location	Number of archival pools (date)	Number of recent pools (date)	Total
*Aedeomyia catasticta*	NT, WA	15 (1973–76)	1 (2018)	16
*Aedes alboannulatus*	WA	7 (1988–90)	2 (2014)	9
*Aedes alboscutellatus*	NT		2 (2018)	2
*Aedes camptorhynchus*	WA	63 (1988–91)	19 (2014)	82
*Aedes clelandi*	WA	11 (1990)	1 (2014)	12
*Aedes hesperonotius*	WA	1 (1990)	2 (2014)	3
*Aedes kochi*	NT		3 (2018)	3
*Aedes normanensis*	NT, WA		355 (2011–14)	355
*Aedes notoscriptus*	NT, WA	2 (1990)	3 (2014–18)	5
*Aedes ratcliffei*	WA	7 (1990)		7
*Aedes* species	WA		1 (2014)	1
*Aedes turneri*	WA		1 (2014)	1
*Aedes vigilax*	NT, WA		9 (2011–14)	9
*Anopheles amictus*	NT		6 (2013)	6
*Anopheles annulipes*	NT, WA	3 (1990)	29 (2013–14)	32
*Anopheles atratipes*	WA	1 (1990)		1
*Anopheles bancroftii*	NT		9 (2018)	9
*Anopheles meraukensis*	QLD	5 (2000–01)		5
*Coquillettidia* species	WA	4 (1990)		4
*Coquillettidia xanthogaster*	NT		13 (2018)	13
*Culex annulirostris*	NSW, NT, PNG, QLD, WA	67 (1990–2007)	66 (2011–18)	133
*Culex australicus*	WA	6 (1988–90)	2 (2014)	8
*Culex bitaeniorhynchus*	NT		2 (2013–18)	2
*Culex gelidus*	NT		12 (2018)	12
*Culex globocoxitus*	WA	7 (1988–90)	3 (2014)	10
*Culex pullus*	NT, WA		16 (2011–18)	16
*Culex quinquefasciatus*	NT, WA	3 (1990)	11 (2014–18)	14
*Culex* species	WA	1 (1990)	3 (2011–14)	4
*Culex vishnui* group	NT		7 (2018)	7
*Culiseta atra*	WA	6 (1990)	1 (2014)	7
*Mansonia uniformis*	NT, WA		19 (2011–18)	19
*Uranotaenia albescens*	NT		3 (2018)	3
*Verrallina funerea*	NT		1 (2018)	1
**TOTAL**		209	602	811

### Vector competence of *Culex* mosquitoes for BgV

To determine whether this vertebrate-restricted virus could be horizontally transmitted by field collected mosquitoes, laboratory reared progeny of wild *Cx*. *annulirostris* and *Cx*. *sitiens* collected from Cairns, northern Queensland, were exposed to BgV using methods previously published [35]. After being exposed to an infectious blood-meal with a titre of 10^7^ TCID_50_ infectious units per milliliter (TCID_50_ IU/mL), infection rates were 11.3% (8/71) and 18.8% (3/16) for *Cx*. *annulirostris* and *Cx*. *sitiens*, respectively (Table 2 and Table 3). BgV was detected in the legs+wings of 8/8 positive blood-fed *Cx*. *annulirostris* mosquitoes whilst none of the three positive *Cx*. *sitiens* mosquitoes had virus disseminated in their legs+wings (Table 2 and Table 3). Half of the positive *Cx*. *annulirostris* had detectable levels of virus in their saliva (4/8), resulting in an overall transmission rate of 5.6% (4/71) for *Cx*. *annulirostris;* there was no detectable transmission for *Cx*. *sitiens* (Table 2 and Table 3). In addition to exposing the mosquitoes to BgV *via* a blood-meal, *Cx*. *annulirostris* and *sitiens* mosquitoes were injected intrathoracically with the virus, in order to bypass the midgut infection and escape barriers, and to provide a group with a controlled virus dose. Injecting BgV intrathoracically achieved 100% infection and dissemination rates (45/45 for *Cx*. *annulirostris* and 11/11 for *Cx*. *sitiens*). The virus was found in high prevalence in the saliva of injected mosquitoes, with 82.2% (37/45) and 81.8% (9/11) transmission rates for *Cx*. *annulirostris* and *Cx*. *sitiens* respectively (Table 2 and Table 3).

**Table 2:** Infection, dissemination and transmission rates of BgV in injected (IT) and bloodfed (oral) *Cx*. *annulirostris* mosquitoes.

Mode of exposure	Day post exposure	% Infection^a^	% Dissemination^b^	% Dissemination /infection^c^	% Transmission^d^	% Transmission /dissemination^e^
IT	8	100 (9/9)	100 (9/9)	100 (9/9)	66.7 (6/9)	66.7 (6/9)
IT	10	100 (36/36)	100 (36/36)	100 (36/36)	86.1 (31/36)	86.1 (31/36)
Oral	13	11.3 (8/71)	11.3 (8/71)	100 (8/8)	5.6 (4/71)	50 (4/8)

^a^Percentage of mosquitoes containing virus in their bodies (number positive/number tested)

^b^Percentage of mosquitoes containing virus in their legs+wings (number positive/number tested)

^c^Percentage of infected mosquitoes containing virus in their legs+wings (number positive/number infected)

^d^Percentage of mosquitoes containing virus in their expectorate collected in capillary tubes (numbers positive/number tested)

^e^Percentage of mosquitoes with a disseminated infection containing virus in their expectorate collected in capillary tubes (number positive/number disseminated)

**Table 3:** Infection, dissemination and transmission rates of BgV in injected (IT) and bloodfed (oral) *Cx*. *sitiens* mosquitoes.

Mode of exposure	Day post exposure	% Infection^a^	% Dissemination^b^	% Dissemination /infection^c^	% Transmission^d^	% Transmission /dissemination^e^
IT	8	100 (11/11)	100 (11/11)	100 (11/11)	81.8 (9/11)	81.8 (9/11)
Oral	11	22.2 (2/9)	0 (0/9)	0 (0/2)	0 (0/9)	N/A
Oral	13	14.3 (1/7)	0 (0/7)	0 (0/1)	0 (0/7)	N/A

^a^Percentage of mosquitoes containing virus in their bodies (number positive/number tested)

^b^Percentage of mosquitoes containing virus in their legs+wings (number positive/number tested)

^c^Percentage of infected mosquitoes containing virus in their legs+wings (number positive/number infected)

^d^Percentage of mosquitoes containing virus in their expectorate collected in capillary tubes (numbers positive/number tested)

^e^Percentage of mosquitoes with a disseminated infection containing virus in their expectorate collected in capillary tubes (number positive/number disseminated)

**Fig 1:**
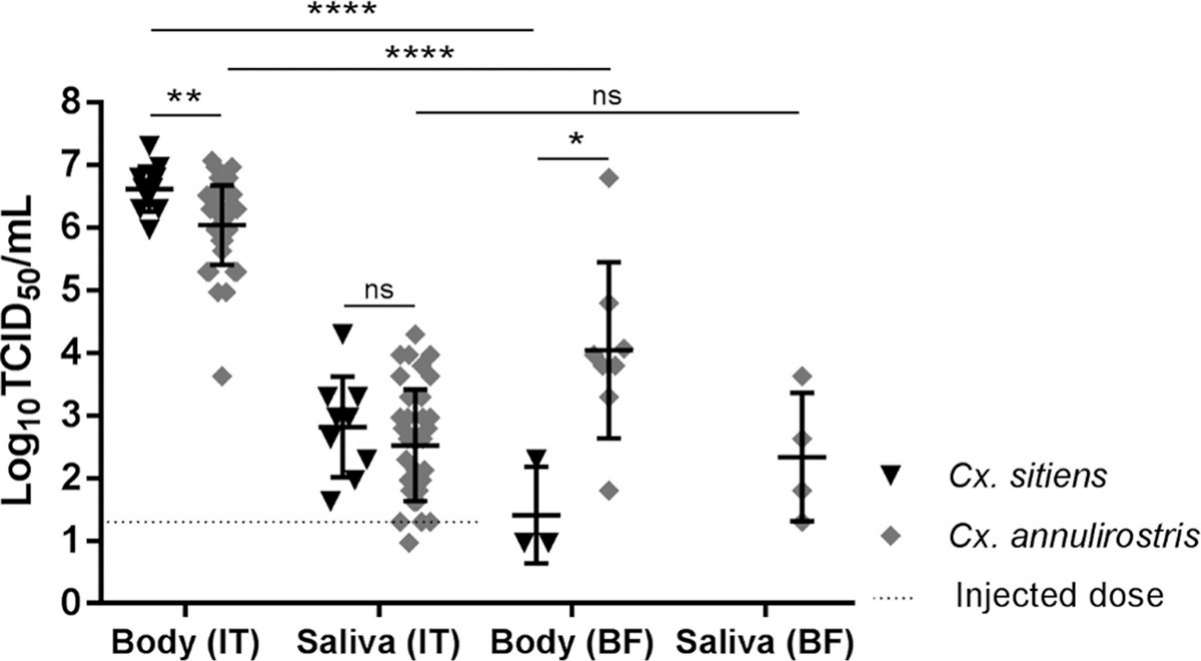
BgV titres in intrathoracically injected (IT) and blood-fed (BF) mosquito bodies and saliva. Error bars represent the standard deviation and middle bars represent the mean. The results were analysed with a parametric unpaired t-test. ns = not significant (P > 0.05), * = P < 0.05, ** = P < 0.01, **** = P < 0.0001.

The level of BgV amplification in *vivo* was measured in injected and bloodfed mosquitoes. Whilst the mosquitoes were injected with the equivalent of approximately 20 TCID_50_ IU, body titres of 10^6^ TCID_50_ IU per injected *Cx*. *annulirostris* were recovered on average eight to ten days post exposure, and 10^6.6^ TCID_50_ IU per injected *Cx*. *sitiens*, which indicated successful replication of the virus in the mosquito tissues (Fig 1). There was a significant difference between the titres in the bodies of blood-fed *Cx*. *sitiens* and *Cx*. *annulirostris*, with a higher titre in *Cx*. *annulirostris* (*P* = 0.0149). This was also the case in the bodies of injected mosquitoes, with slightly higher titres on average detected in *Cx*. *sitiens* (*P* = 0.0058). Titres were significantly lower in the mosquito bodies of the blood-fed mosquitoes than in the injected mosquitoes (*P* < 0.0001 for both species). In contrast, virus titres in saliva expectorates of injected mosquitoes were not significantly different between *Cx*. *sitiens* and *Cx*. *annulirostris* (*P* = 0.3712). Similarly, titres in the saliva were not significantly different between injected and blood-fed *Cx*. *annulirostris*, although this could be the result of a longer extrinsic incubation for bloodfed mosquitoes (*P* = 0.6977).

### Tissue tropism in infected *Culex* mosquitoes

**Fig 2:**
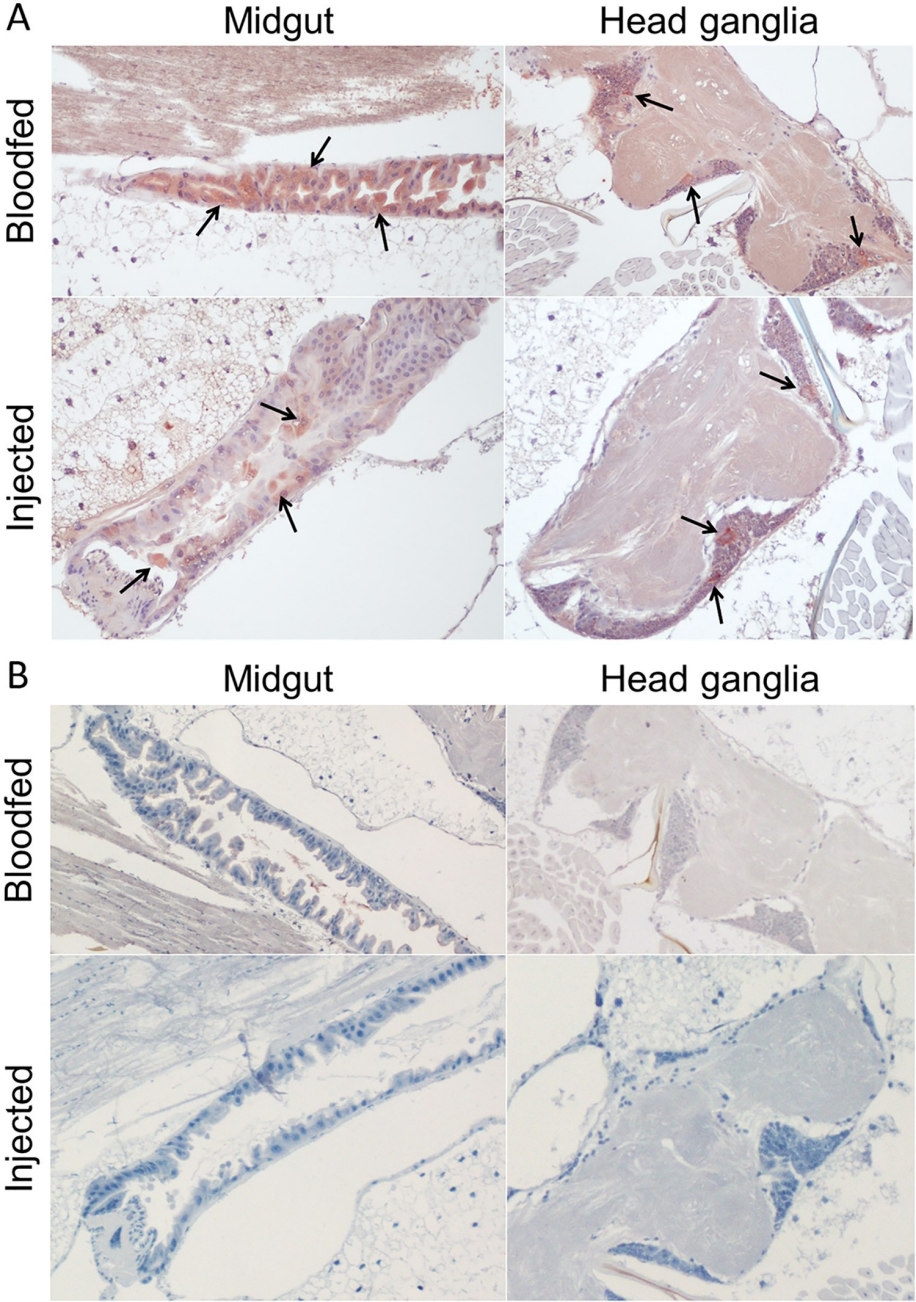
Detection of BgV in blood-fed and injected *Cx*. *annulirostris* infected with BgV using IHC. The mosquito sections were immunolabeled using a cocktail of BgV-reactive mAbs which enabled identification of (A) positive mosquitoes showing presence of BgV antigen in red (arrows) in the midgut epithelial cells and the neuronal cells in the head ganglia, and negative mosquitoes (B) with no specific signal.

BgV was detected by IHC in the midgut epithelial cells and neuronal cells of 8/25 bloodfed (harvested 14 days post-infection) and 20/25 injected *Cx*. *annulirostris* (harvested 10 days post-infection). There was more virus antigen present in the midgut of blood-fed mosquitoes, while injected mosquitoes displayed more antigen in the neuronal cells (Fig 2). Viral antigen was also detected in the fat bodies proximal to the gonads and in the salivary glands.

### BgV inhibits replication of pathogenic flaviviruses *in vitro*

**Fig 3:**
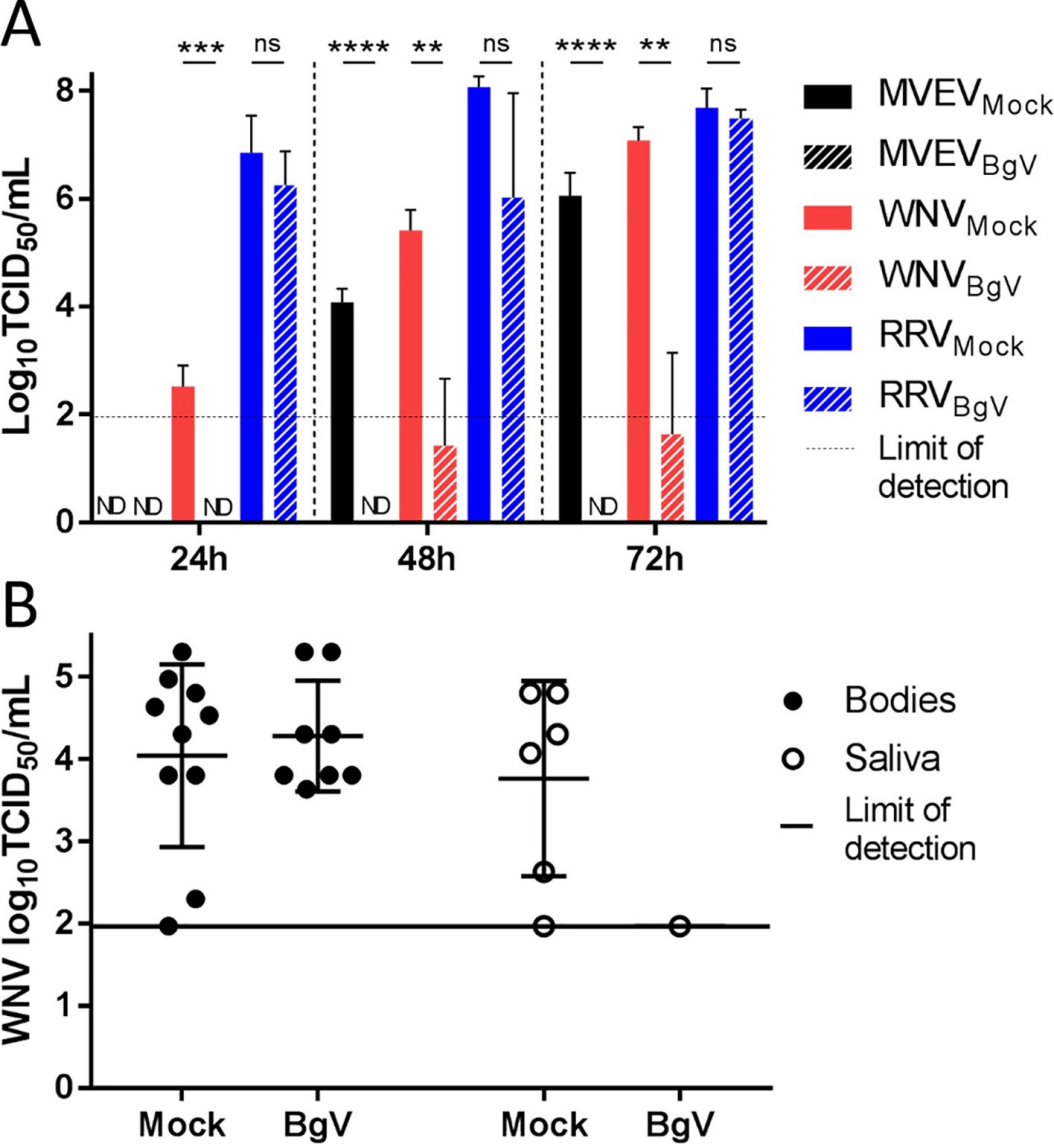
Super-infection interference *in vitro* and *in vivo*. (A) Viral titres of super-infecting viruses MVEV, WNV and RRV in C6/36 cells with primary mock or BgV infection. The super-infecting viruses were titrated on Vero cells to prevent further interference from BgV. ND = not detected. Error bars stand for the standard deviation. The results were analysed with a parametric unpaired t-test. ns = not significant (P > 0.05), ** = P < 0.01, *** = P < 0.001, **** = P < 0.0001. (B) WNV titres in the bodies and saliva of *Culex* mosquitoes mock- or BgV-injected, and subsequently exposed to WNV in an infectious blood-meal. Error bars represent the standard deviation and middle bars represent the mean.

To determine whether prior infection with BgV affects replication of pathogenic flaviviruses, mosquito C6/36 cells were either mock- or BgV-infected for five days, and subsequently infected with RRV, MVEV or WNV. BgV-infected cells were shown to be significantly less permissive to WNV and MVEV super-infection than mock-infected cells (Fig 3A). Indeed, no MVEV replication could be detected in BgV infected cells at any time points, while the MVEV titre increased over time in mock-infected cells. The difference was statistically significant at 48h and 72h with *P* < 0.0001. Significantly lower titres were attained for WNV in BgV infected cells at all time points, with *P* = 0.0003 at 24h, *P* = 0.0061 at 48h and *P* = 0.0035 at 72h. This super-infection interference phenomenon was not observed for RRV (with *P* = 0.3252 at 24h, *P* = 0.1427 at 48h and *P* = 0.4145 at 72h), suggesting BgV specifically interfered with flavivirus replication *in vitro*.

### BgV interferes with transmission of WNV *in vivo*

To examine whether BgV interfered with infection and transmission of pathogenic flaviviruses *in vivo*, Cx. *annulirostris* mosquitoes were injected with either growth medium or BgV (approximately 220nL at 10^5^ TCID_50_ IU/mL). Seven to eight days later, the mosquitoes were offered a blood/virus mixture containing 10^6^ TCID_50_ IU/mL of WNV. At seven days post-infection, no mosquito bodies tested positive for WNV in either group (n = 29 mock-injected and n = 23 BgV-injected), while 15/23 BgV-injected mosquitoes had detectable levels of BgV. At ten days post-infection, there was no significant difference in the number of mosquito bodies positive for WNV between mock- (10/31) and BgV-infected (8/27) mosquitoes (*P* > 0.9999), and the titres were not significantly different (*P* = 0.6019) (Table 4, Fig 3B). Similarly, there was no significant difference in WNV dissemination rates between the two groups (9/10 mock-injected and 8/8 BgV-injected) (*P* > 0.9999) (Table 4). However, fewer mosquitoes had detectable amounts of WNV in their saliva in the BgV-infected group (1/8) compared to the mock-infected group (6/10) (Table 4), although the difference was not significant (*P* = 0.0656). The single BgV-infected mosquito with WNV detected in saliva had a WNV saliva titre at the limit of detection, (10^1.97^ TCID_50_ IU/mL), which was lower than the average for WNV-positive saliva of mock-injected mosquitoes (10^4.42^ TCID_50_ IU/mL) (Fig 3B).

**Table 4:** Infection, dissemination and transmission rates of WNV and BgV in *Culex* mosquitoes primarily mock- or BgV-injected and super-infected with WNV *via* bloodfeeding. NT = Not tested.

Primary	Virus tested	Day post exposure	% Infection^a^	% Dissemination^b^	% Dissemination /WNV infection^c^	% Transmission^d^	% Transmission /WNV dissemination^e^
**BgV**	BgV	7	65.2 (15/23)	NT	NT	NT	NT
	WNV	7	0 (0/23)	NT	NT	NT	NT
**Mock**	WNV	7	0 (0/29)	NT	NT	NT	NT
**BgV**	BgV	10	100 (27/27)	NT	100 (8/8)	NT	87.5 (7/8)
	WNV	10	29.6 (8/27)	29.6 (8/27)	100 (8/8)	3.7 (1/27)	12.5 (1/8)
**Mock**	WNV	10	32.3 (10/31)	29.0 (9/31)	90 (9/10)	19.4 (6/31)	60 (6/10)

^a^Percentage of mosquitoes containing virus in their bodies (number positive/number tested)

^b^Percentage of mosquitoes containing WNV in their legs+wings (number positive/number tested)

^c^Percentage of infected mosquitoes containing virus in their legs+wings (number positive/number WNV infected)

^d^Percentage of mosquitoes containing WNV in their expectorate collected in capillary tubes (numbers positive/number tested)

^e^Percentage of mosquitoes with a disseminated infection containing virus in their expectorate collected in capillary tubes (number positive/number WNV disseminated)

## Discussion

The results from experiments performed in *Culex* mosquitoes provide evidence that BgV is likely transmitted and maintained in the environment using a classical arbovirus transmission cycle. This cycle comprises ingestion of an infectious blood-meal, replication in various tissues and transmission *via* the saliva of the infected mosquito. The localization of BgV in various mosquito tissues was consistent with the classical model for flavivirus dissemination in mosquitoes but in contrast to what we have previously reported for Australian insect-specific flaviviruses, which appear to be restricted to the midgut of their mosquito hosts [19, 26]. Despite this confirmation that BgV could be part of a classical arbovirus transmission cycle, the relatively low proportion of *Cx*. *annulirostris* mosquitoes which could transmit the virus (5.6%) suggested that *Culex* mosquitoes were not highly competent vectors of BgV as they are of other vertebrate-infecting flaviviruses such as WNV or JEV (> 50% transmission rate for both) in Australia [3, 17, 43]. Indeed, *Cx*. *annulirostris* and *Cx*. *sitiens* appeared to express a midgut infection barrier, with less than 25–30% of mosquitoes infected following ingestion of an infectious blood-meal of BgV. *Cx*. *annulirostris* did not appear to express a midgut escape barrier, as all infected mosquitoes had a disseminated infection and 50% of these transmitted the virus. Although numbers were relatively low, none of the infected *Cx*. *sitiens* had a disseminated infection, suggesting the presence of a midgut escape barrier in this species. Further evidence for the presence of these various barriers was provided by the results of the IT inoculations, whereby all mosquitoes from both species possessed a disseminated infection and the majority of these transmitted the virus. The viral titres recovered from the injected mosquito samples (bodies and saliva) were similar to what has been found for WNV-injected *Culex* mosquitoes [26], while the titres in the blood-fed mosquito samples (bodies and saliva) were lower than for WNV in *Culex* mosquitoes [26, 43].

We conducted our *in vivo* experiments with *Cx*. *annulirostris* and *Cx*. *sitiens*, as these species belong to the *Cx*. *sitiens* group, which are the only taxonomic group that have yielded detections of BgV. It is possible that members of other mosquito genera may be more efficient vectors of BgV and may play a greater role in maintaining the virus in nature. Indeed, the infection and transmission rates of *Culex*-associated flaviviruses such as WNV or JEV are much lower in *Aedes* mosquitoes (<35% infection; <15% transmission and 27% infection; 25% transmission respectively) than in *Culex* (>70% infection; >50% transmission and >90% infection; >50% transmission respectively), and more similar to what was observed for BgV in *Culex* [3, 17]. Additionally, the virus may have been present in other genera during the time these collections were undertaken, but remained undetected. The preponderance for BgV to be detected only in *Cx*. *sitiens* subgroup mosquitoes could be an artefact of the emphasis placed on processing *Culex spp*. which are the primary vectors of JEV, during investigations of this virus in northern Australia [29]. Other genera were discarded and so were underrepresented in the original study that yielded BgV. Further evidence that BgV may be transmitted by other mosquito species is provided by the phylogenetic position of BgV in the YFV group, in which the viruses are thought to be *Aedes*-associated rather than *Culex*-associated. Finally, in immuno-assays with anti-dsRNA mAbs, BgV can only be detected in cells that have been fixed in 100% acetone, as opposed to our standard fixative buffer with 20% acetone for ELISA, which has previously been demonstrated for DENV serotypes 1 and 2, indicating that BgV may share a similar mode of replication with these two *Aedes-*associated flaviviruses *in vitro* [28]. Collectively, this suggests that even though BgV can be horizontally transmitted by *Culex* mosquitoes and has only ever been isolated from *Culex* mosquitoes, it might be preferentially *Aedes*-associated rather than *Culex*-associated. Clearly, further *in vivo* experiments with other genera, particularly *Aedes* spp., are needed to incriminate other mosquito species that could serve as vectors of BgV.

We attempted to address the underrepresentation of other mosquito genera by screening an additional 811 archival and recent pools from a wide range of species, collected from a broad geographical range in Australia and Papua New Guinea. No BgV isolates could be recovered from these pools, despite the tested mosquitoes being from a wide geographical (Australian states of New South Wales, Queensland, Northern Territory, Western Australia, as well as Papua New Guinea) and temporal (1973 to 2018) distribution. The low prevalence observed for BgV in Australasian mosquitoes is not unusual for a vertebrate-infecting flavivirus [44, 45], with many thousands of mosquitoes often yielding a single positive sample only. This low prevalence is reflective of the intricate virus transmission cycle between specific amplifying vertebrate hosts and competent mosquito vectors. Such a low number of positive samples, found only in one mosquito species and one location, *Cx*. *annulirostris* from Cape York Peninsula, indicates a small ecological niche for this virus. It is possible that BgV has a cryptic vertebrate host found only in certain parts of Australia, which would also fit with its apparent host-restriction in some vertebrate cells [18]. There could also be a discrepancy between the optimal amplifying vertebrate host of BgV and the feeding preferences of its optimal mosquito vector, resulting in low incidence rates in Australasian mosquitoes. Indeed, the most competent mosquito vector species may not include the best BgV amplifying vertebrate host in its blood feeding patterns. This phenomenon has been shown to potentially reduce JEV transmission in certain areas, as *Cx*. *annulirostris* preferentially feeds on cattle and wallabies, while the virus is not amplified to sufficient levels in these animals to be transmitted [45, 46]. It should however be noted that the sample size for each mosquito population tested here was relatively small. Thus, further assessment of the prevalence of BgV in mosquito species collected in the Cape York region should be undertaken, along with assessment of the prevalence of BgV-specific antibodies in major vertebrate species in the area, such as agile wallabies, in order to confirm the primary vector and vertebrate host of BgV. These considerations will help better understand its ecology, host-restriction, evolution and potential to emerge as a virus capable of causing disease in humans or other animals.

Considering that flaviviruses have previously been shown to interfere with the replication of related viruses *in vitro* and *in vivo*, and that BgV was isolated in a cohort of samples that also yielded several other flavivirus isolates, super-infection interference studies were performed [18, 29]. In our laboratory setting, it was clear that BgV could interfere with the replication and transmission of pathogenic flaviviruses both in C6/36 *Ae*. *albopictus* cells *in vitro* and in *Culex* mosquitoes *in vivo*. The data generated here demonstrated that primary infection with BgV completely prevented replication of MVEV in C6/36 cells, strongly suppressed replication of WNV, but had no significant effect on the alphavirus RRV, suggesting the interference was specific to flaviviruses. Additionally, it was shown that while BgV did not seem to interfere with WNV replication or dissemination in *Culex* mosquitoes, WNV transmission was inhibited in the presence of BgV. Indeed, there was no difference in the number of mosquitoes positive for WNV or in the WNV titres in the bodies of mock- and BgV-injected WNV-blood-fed mosquitoes. These results suggested that for BgV, the midgut was not the main site for interference. However, it was clear that BgV interfered with WNV after the virus had escaped the midgut barrier, since the number of WNV-positive mosquito saliva and the WNV titre in these saliva samples were lower in BgV-injected mosquitoes than in mock-injected mosquitoes. These data were similar to what was found with lineage II insect-specific flavivirus, Nhumirim virus, interfering with WNV in *Cx*. *quinquefasciatus* mosquitoes [47], but differ from what was found for Australian lineage I insect-specific flavivirus Palm Creek virus, which seemed to interfere with WNV replication in the midgut epithelial cells of infected mosquitoes [26]. However, it should be noted that in each study, intrathoracic inoculation was used to infect the mosquitoes with the primary virus, in this case BgV. While it was shown that BgV was present in detectable levels in the midgut epithelial cells of injected mosquitoes (Fig 2), more BgV was present in the midgut of mosquitoes orally infected with BgV. This is consistent with the midgut epithelium being the first cells encountered by a virus in an infectious blood-meal. Thus, mosquitoes orally infected with BgV, presumably the natural route of infection, may provide more resistance to subsequent infection of the midgut. In most instances and in accordance with our *in vitro* data, reports suggest that flaviviruses do not interfere with the replication of viruses from other families or genera such as alphaviruses, parvoviruses, or baculoviruses [48–54]. However, other authors have reported that an interfering interaction between flavivirus and other virus families such as rhabdoviruses, parvoviruses or alphaviruses can occur [55–58]. These discrepancies are most likely due to experimental design differences, such as the virus species used, the order of infections or whether the experiment is a co-infection or a super-infection. Even though these interactions can happen in laboratory models, *in vitro* or *in vivo*, they may not actually occur in nature, since the prevalence of arthropod-borne viruses in mosquito populations is quite low, as mentioned above.

In conclusion, we have successfully shown that the flavivirus BgV can be transmitted horizontally by *Cx*. *annulirostris*, a member of the *Cx*. *sitiens* subgroup, from which all BgV isolates have been obtained, and thereby be included in a classical arbovirus transmission cycle. We have also demonstrated that BgV can interfere with *Culex-*associated WNV transmission *in vivo*. Further investigations could include vector competence studies of *Aedes* mosquitoes for BgV to draw comparisons with the data presented here, as well as super-infection interference experiments with *Aedes*-associated flaviviruses. Future work should also comprise studying the other side of the transmission cycle: the host-restriction of BgV in vertebrates. Overall, the presented data contribute to elucidating the ecology of an Australian flavivirus and help to further the understanding of mechanisms of interference between flaviviruses in mosquitoes.
